# The association between Helicobacter pylori and obesity: a systematic review and meta-analysis of case–control studies

**DOI:** 10.1186/s40842-021-00131-w

**Published:** 2021-07-10

**Authors:** Ali Baradaran, Hojat Dehghanbanadaki, Sara Naderpour, Leila Mohammadi Pirkashani, Abdolhalim Rajabi, Roya Rashti, Sevda Riahifar, Yousef Moradi

**Affiliations:** 1 Endocrine Research Center, Institute of Endocrinology and Metabolism, Iran University of Medical Sciences, Tehran, Iran; 2 Students Scientific Research Center, Tehran University of Medical Sciences, Tehran, Iran; 3 Department of Epidemiology, School of Public Health, Iran University of Medical Sciences, Tehran, Iran; 4 Clinical Research Development Center, Imam Reza Hospital, Kermanshah University of Medical Sciences, Kermanshah, Iran; 5 Department of Health Management and Social Development Research Center, Faculty of Health, Golestan University of Medical Sciences, Gorgan, Iran; 6 Social Determinants of Health Research Center, Research Institute for Health Development, Kurdistan University of Medical Sciences, Sanandaj, 66179-13446 Iran; 7 Department of Biostatistics, Faculty of Public Health, Iran University of Medical Sciences, Tehran, Iran

**Keywords:** Helicobacter pylori, Obesity, Case–control, Systematic review, Meta-analysis

## Abstract

**Introduction:**

The relationship between H. pylori infection and obesity development has remained controversial among various studies. The aim of this study was to clarify the pooled effect of H. pylori infection on the development of obesity and vice versa.

**Methods:**

We searched international databases including Medline (PubMed), Web of sciences, Scopus, EMBASE, Cochrane, Ovid, and CINHAL to retrieve all case–control studies reporting the effect of H. pylori on obesity and vice versa, which had been published in English between January 1990 and June 2019. The quality of included studies was assessed by the Modified Newcastle–Ottawa Scale for Case–Control studies. The logarithm of the odds ratio (OR) and its standard error was used for the meta-analysis.

**Results:**

Eight case–control studies with 25,519 participants were included for qualitative and quantitative analyses. The pooled analysis showed that obese participants had a higher risk of H. pylori infection than lean participants with an odds ratio of 1.46 (95%CI: 1.26, 1.68). Also, the pooled analysis revealed that participants infected by H. pylori had a higher risk of obesity than non-infected participants with an odds ratio of 1.01 (95%CI: 1.01, 1.02).

**Conclusion:**

The results of this meta-analysis showed that there was a positive correlation between the risk of H. pylori infection and the prevalence of obesity development. Thus, H. pylori positive patients were more likely to be obese, and obese individuals had higher risks of H. pylori infection.

## Introduction

Obesity as a chronic non-communicable disease has become a significant public health concern around the world, which can result in or exacerbate a high burden of health conditions like hypertension, hyperlipidemia, cardiovascular diseases, diabetes mellitus, chronic kidney diseases, fatty liver diseases, etc. Besides, its prevalence has increased during recent years, probably due to modern lifestyles, less physical activity, high-calorie intake, and so on [1–4]. Recently, animal and human studies revealed that gut microbiota could balance the energy hemostasis of the host and subsequently influence the development of obesity [5, 6]. In a retrospective study on the Chinese population, the results showed that the prevalence of Helicobacter pylori infection in obese people was higher than that in non-obese people, but the results were not statistically significant [7]. In a meta-analysis of the Chinese population, the results showed that people with Helicobacter pylori infection had an odds ratio of 1.20 times higher than those without infection [8]. Also, a meta-analysis study conducted to determine the exact prevalence of Helicobacter pylori infection, showed that the prevalence of this infection in the world was 44.3%, which was higher in men than women [9].

In this subject, the correlation between obesity and *Helicobacter pylori* (H. pylori) has been evaluated in many studies [10–12]. H. pylori is a spiral-shaped, non-spore-forming and gram-negative bacterium residing on the epithelium of the human stomach. It has been reported that 4.4 billion people around the world were infected with this pathogen in 2015, who approximately included the half of the world [9]. This kind of bacteria affects human health through developing various diseases, e.g. peptic ulcer diseases, gastric cancer, and mucosa-associated lymphoma [13–15]. However, the relationship between H. pylori infection and obesity development has been controversial between various studies [10–12, 16, 17]. So far, various studies have been done, both as a case study and as a cohort, to investigate the relationship between the presence of Helicobacter pylori infection and the occurrence of obesity or vice versa, but contradictory results of these studies have been published. Some studies showed that people with Helicobacter pylori infection had a higher body mass index than people without infection. An Israeli study found that people with Helicobacter pylori infection were more obese than healthy people while another study found no link between infection and the occurrence of obesity [18]. Lender N et al. also found in a review study that there was no association between obesity and the prevalence of Helicobacter pylori infection. In a study on 3,578 people aged 16 to 64 years, the results showed that the presence of Helicobacter pylori infection increased the metabolic syndrome incidence in patients so that the risk was higher in women than men [14, 19, 20]. According to the statements above, no meta-analysis study has been performed in the world to determine the association between Helicobacter pylori infection and obesity or vice versa. Conducting such a study, in addition to achieving the appropriate effect size to find a causal relationship, can provide suitable information to health policy makers and clinicians to take appropriate preventive measures and timely treatment. Therefore, the current meta-analysis aimed to clarify the pooled effect of H. pylori infection on the development of obesity and vice versa.

## Methods

This systematic review and meta-analysis were in accordance with the guidelines of Preferred Reporting Items for Systematic Reviews and Meta-Analyses (PRISMA) for reviews of analytical observational studies (case–control) [21–23].

### Search strategy and screening

**Fig. 1:**
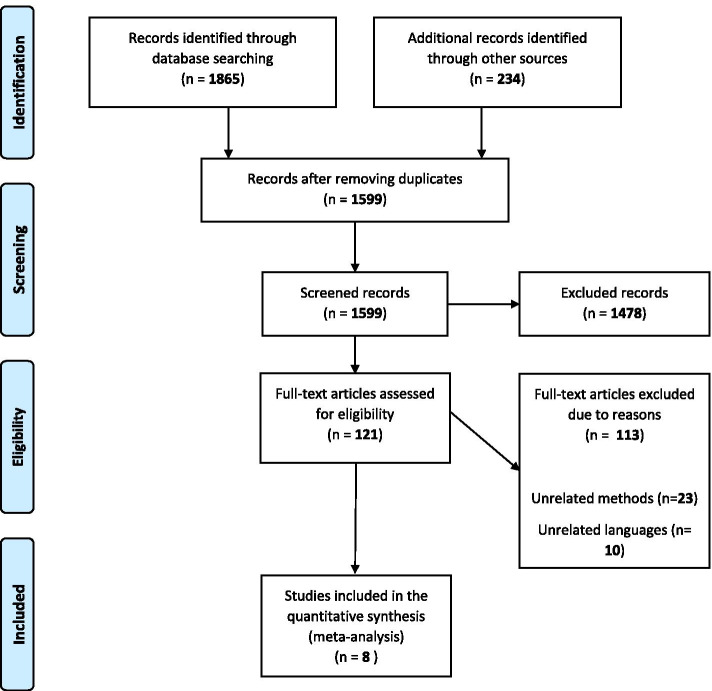
The flow diagram of the literature search and study selection

We searched Medline (PubMed), Web of sciences, Scopus, EMBASE, Cochrane, Ovid, and CINHAL for original articles published in English from January 1990 to December 2019. We used the following index terms and keywords: “Helicobacter pylori”, “Campylobacter pylori”, “Obesity”, “Hypoventilation Syndrome”, “Obesity-Hypoventilation Syndrome”, “Abdominal Obesities”, “Central Obesity”, “Visceral Obesity”, “Benign Obesity Metabolically Healthy Obesity”, “Metabolically Benign Obesity”, “Healthy Obesity”, “Severe Obesities”, “Morbid Obesity”, “Body Mass Index”, “Overweight”, and “Quetelets Index”. After removing duplicated articles from the primary search results, two independent reviewers (SN and AB) screened the title and abstract of the remaining articles and eliminated the irrelevant ones. Any disagreement between them was discussed and if no consensus was achieved, the conflict was resolved by the third researcher (YM). The PRISMA flowchart of this study was shown in Fig. 1.

### Eligibility criteria

The articles met the following criteria, were included in this systematic review: (1) case–control studies, (2) human populations, (3) study populations were patients with BMI ≥ 25, and (4) Helicobacter pylori infection was an independent variable. Besides, the exclusion criteria were case reports, reviews, animal studies, letters to the editor, and cohort studies.

### Data extraction

A data extraction form was created based on our group discussion according to 10 different types of the study. Then, it was modified and used by the data extractors. Two researchers (AB and SN) independently extracted and entered data into the modified extraction form. Any disagreement was assessed by both and if a consensus was not reached, a third researcher (YM) would make a decision. The following information was extracted from the included studies: 1) the name of the first author, 2) the date of publication, 3) the country, 4) control subjects, 5) study populations, 6) the age, 7) the mean of BMI, 8) measurement of association, 11) controlled variables, and 12) the method of bacteria detection.

### Quality assessment or risk of bias

All studies were assessed by the Modified Newcastle–Ottawa Scale for case–control studies [24]. Using this tool, each study was judged in terms of eight items, and categorized into three groups: the selection of the study groups; the comparability of the groups; and how to measure exposure and the desired outcome in the selected case–control studies [25]. Two researchers (YM and AB) independently evaluated the quality of included studies and the results were reported in Table 1.

**Table 1:** The main characteristics of case–control studies on the effect of H. pylori infection on the risk of obesity and vice versa

Authors	Control subjects (selection methods)	Study population	Age	Sample size	Obesity (mean BMI)	Measurement of association, odds ratio (CI 95%)	Controlled variables	Bacteria detection	NOS score
**Ali M. Al-zubaidi, et al. (2018) (Saudi Arabia) **[21]	Non-obese (BMI < 30) who underwent endoscopy	Obese and non-obese	Case: 31.51 ± 8.27 Control: 30.90 ± 7.93	680 Case: 340 Control:340	BMI ≥ 30	1.98 (1.45–2.70)	Age-sex	Histology or biopsy	7
**Nahum Méndez-sánchez, et al. (2008) (Mexico) **[24]	Non H. pylori	Chronic gastritis and H. pylori (mild, moderate, severe) and chronic gastritis without H. pylori	Case: 48.16 ± 16. 44 Control: 42.88 ± 17.04	283 Case: 189 Control:94	BMI ≥ 30	The density of H. pylori: Presence of infection: 0.88 (0.45 – 1.72) mild:1.04 (0.51–2.09) Moderate: 0.62 (0.21–1.83) Severe: 0.37 (0.46–3.07) Moderate–severe 0.56 (0.21–1.53)	-	Histology or biopsy	6
**Ming-shiang Wu, et al. (2005) (Taiwan) **[8]	normal weight (BMI < 25)	Obese patients of BMI ≥ 35 with serious comorbidity or a BMI ≥ 40 and control BMI < 25	Case: 31.9 ± 9.2 Control: 32.3 ± 9.5	1097 Case: 414 Control:683	BMI ≥ 35 BMI ≥ 40	0.50 (0.39 – 0.65)	Geographical area, socioeconomic status	Histology	7
**Erol Arslan, et al. (2009) (Turkey) **[9]	Normal weight (BMI < 25)	Obese and Non-obese	Case: 24.3 ± 5.4 Control: 25.5 ± 5.4	214 Case: 103 Control:111	Mean BMI Case: 34.6 ± 3.7 Control:24.2 ± 2.8	2.11 (1.49 – 3.00)	Geographical area, socioeconomic status	One step H. pylori test device	7
**Chengfu Xu, et al. (2004) (China) **[26]	Non-H.pylori	Adults with H.pylori who underwent health checkups	Case: 46.0(40.0.53.0) Control: 46.0(39.0–54.0)	8820 Case: 3859 Control: 4961	Mean BMI Case: 24.01 (21.77–26.23) Control: 23.63(21.52 – 2581)	1.018 (1.011–1.025)	Age – sex- BMI—waist circumference—systolic blood pressure—diastolic blood pressure—alanine aminotransferase -	^13^C-urea breath tests	6
**Basit Siddiqui, et al. (2018) (Pakistan) **[25]	Normal weight(BMI:18.5 – 23)	Adults who attended the gastroenterology clinic for dyspeptic symptoms that included abdominal discomfort or pain, bloating, and nausea, and underwent gastroscopy	Mean age: 44 ± 16 Case: 46.0(40.0.53.0) Control: 46.0(39.0–54.0)	698 Case: 399 Control: 299	BMI > 28	2.91 (2.01- 4.20)	-	Biopsy, Histological examination, ^13^C-urea breath test, H. pylori stool antigen test	7
**G. N. Ioannou, et al. (2004) (USA) **[23]	H. pylori and Cag A Antibodies(-/-)	H. pylori and Cag A Antibodies(-/-), ( ±) and (+ / +)	20 ≥	Case: H. pylori and Cag A ( ±): 1445 (+ / +): 2149 Control: (-/-): 3130	BMI > 30	H. pylori/ Cag A Antibody status (+ / +): 1.2 (0.9–1.6) H. pylori/ Cag A Antibody status ( ±): 1.1 (0.8–1.5)	Ethnicity, age, gender, poverty index, educational Attainment, household crowding index, alcohol consumption, coffee Consumption, country of birth, occupation, geographical region and Metropolitan region,	Elisa	7
**Ilseung Cho, et al. (2005) (USA) **[22]	Negative H. pylori (H. pylori_)	No pregnant participants in the third national health and nutrition examination survey	Mean age: 45.2	H. pylori and Cag A: ( ±) 1,385 (+ / +): 2,634 (H. pylori_): 2,984	BMI > 25	H. pylori/ Cag A Antibody status (+ / +): 1.17 (0.98–1.39) H. pylori/ Cag A Antibody status ( ±): 0.99 (0.80–1.22)	Age, sex, race/ethnicity, alcohol consumption, cigarette smoking, activity level, and years of education	Elisa	7

### Statistical analysis

The logarithm and standard error logarithm odds ratio (OR) were used for the meta-analysis. The DerSimonian and Laird method was used to compute the pooled estimate of odds ratio (OR) with a 95% confidence interval (95% CI) [12]. Because the test for heterogeneity was statistically significant in some analyses, the random effect models were used to estimate the OR. In this study, the w Cochran’s Q test and I^2^ statistic were used to evaluate statistical heterogeneity between studies [13]. Besides, a meta-regression and subgroup analysis were performed to assess the source of heterogeneity between studies. Moreover, publication bias was assessed by the funnel plot and Egger and Begg’s test [14, 15]. Statistical analysis was performed using STATA 16.0 (Stata Corp, College Station, TX, USA).

## Results

### Study selection and characteristics

Our search strategy yielded a total of 1599 possibly relevant articles after the removal of the duplicate ones. Through screening the title and abstract of the retrieved articles, 1478 ones were excluded due to irrelevancy and the remaining 121 articles were reviewed in the full-text for eligibility assessment. Finally, 8 case–control studies [11, 12, 26–31] were selected for meta-analysis by passing the inclusion and exclusion criteria filters (Fig. 1). The general characteristics of the included studies were demonstrated in Table 1. In total, 25,519 participants aged between 24 and 46 years, were included in our meta-analysis. Four studies with 22,830 participants evaluated the impact of obesity on the risk of H. pylori infection while the other four studies with 2689 participants evaluated the effect of H. pylori on the development of obesity. These studies were carried out in different geographical populations consisted of Saudi Arabia, Mexico, Taiwan, Turkey, China, Pakistan, and the USA, between 2004 and 2018. In addition, various techniques were employed to detect the H. pylori infection, including the histology or biopsy (*n* = 4 studies), ^13^C-urea breath test (*n* = 2 studies), ELISA (*n* = 2 studies), H. pylori stool antigen test (*n* = 1 study), and one step H. pylori test device (*n* = 1 study).

### The effect of obesity on the development of H. pylori

**Fig. 2:**
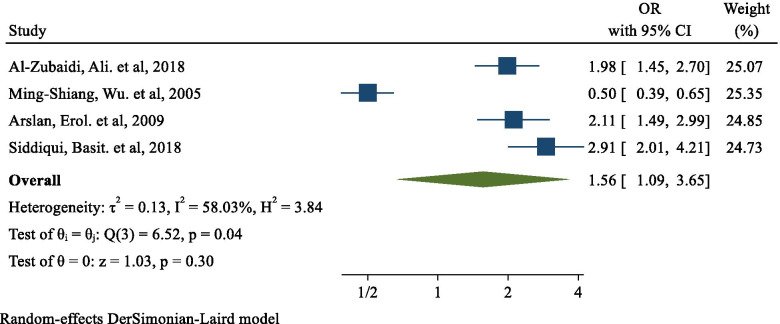
The effect of obesity on the risk of Helicobacter pylori infection

The odds ratio of studies evaluating the effect of obesity on the risk of H. pylori infection ranged between 0.50 (95% CI: 0.39, 0.65) and 2.91 (95% CI: 2.01, 4.21); the pooled analysis of these studies revealed that the risk of H. pylori infection was higher in obese participants than that in lean participants with an odds ratio of 1.56 (95% CI: 1.09, 3.65) and there was low heterogeneity between these studies in estimating the effect of obesity on the risk of H. pylori infection (I-squared = 58.03%, *p* = 0.04) (Fig. 2).

### The effect of H. pylori on the development of obesity

**Fig. 3:**
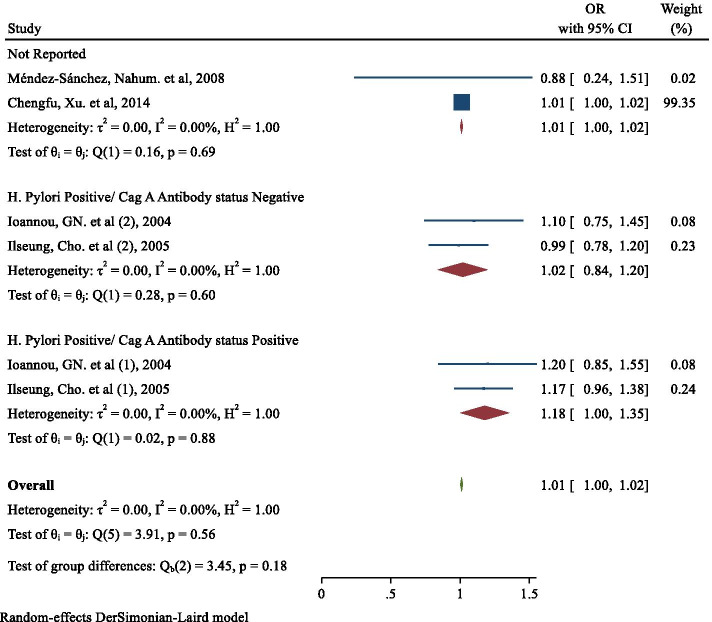
The effect of Helicobacter pylori infection on the risk of obesity

The results showed that if the status of both H. pylori and Cag A Antibody were positive, there was a significant relationship between the presence of H. pylori infection and the occurrence of obesity (OR:1.18; 95% CI: 1.00, 1.35) (Fig. 3). However, in the presence of the positive status of H. pylori and the negative status of Cag A Antibody, the effect of H. pylori infection on the occurrence of obesity was 1.02 (95% CI: 0.84, 1.20), which was not statistically significant (Fig. 3).

### Subgroup analysis of the pooled effect of H. pylori infection on obesity

The subgroup analysis based on the method of diagnosis, would be demonstrated if ELIZA was employed to detect the H. pylori infection. The pooled odds ratio of H. pylori infection on the risk of obesity development was 1.11 (95% CI: 0.99 – 1.25) while the odds ratios of the histology/biopsy technique and ^13^C-urea breath test were 0.88 (95% CI: 0.45 – 1.72) and 1.01 (95% CI: 1.00 – 1.02), respectively. By the way, four studies utilized ELIZA to detect H. pylori infection among the participants and there was no heterogeneity between the results of these studies (I-squared = 0.0%, *p* = 0.627) (Table 2).

**Table 2:** Subgroup analysis of the pooled effect of obesity on H. pylori infection and vice versa based on the diagnosis method

Effect	Subgroup	No. of studies	Summary odds ratio (95% CI)	Between studies			Between subgroups	
				I^2^	P _heterogeneity_	Q	Q	P _heterogeneity_
H. pylori on obesity	Variables adjusted							
	Yes	5	1.01 (1.01 – 1.03)	6.6%	0.372	4.03	4.03	0.0001
	No	1	0.88 (0.45 – 1.72)	-	-	-		
	Method of diagnosis							
	ELIZA	4	1.11 (0.99 – 1.25)	0.0%	0.627	1.76	4.03	0.006
	Histology and biopsy	1	0.88 (0.45 – 1.72)	-	-	-		
	^13^C-urea breath test	1	1.01 (1.00 – 1.02)	0.0%	0.515	1.43		
Obesity on H. pylori	Method of diagnosis							
	Histology and biopsy	3	1.14 (0.96 – 1.36)	40.0%	0.180	1.55	5.14	0.001
	^13^C-urea breath test	1	2.11 (1.48 – 2.99)	-	-	-		
	One step H. pylori test device	1	2.91 (2.01 – 4.20)	-	-	-		

### Subgroup analysis of the pooled effect of obesity on H. pylori infection

On the other hand, the subgroup analysis of the pooled effect of obesity on the risk of H. pylori infection based on the method of diagnosis, revealed that in three studies that had utilized the histology/biopsy approach, obesity didn’t significantly increase the odds of H. pylori infection (odds ratio = 1.14; 95% CI: 0.96 – 1.36) while in other diagnostic methods, e.g. the ^13^C-urea breath test and one step H. pylori test device, obesity increased the odds of H. pylori infection with an odds ratios of 2.11 (95% CI: 1.48 – 2.99) and 2.91 (95% CI: 2.01 – 4.20), respectively. However, it is noteworthy that there was low heterogeneity between studies estimating the effect of obesity on H. pylori infection through the histology/biopsy approach (I-squared = 60.0%, *p* = 0.145) (Table 2).

### Publication bias

**Fig. 4:**
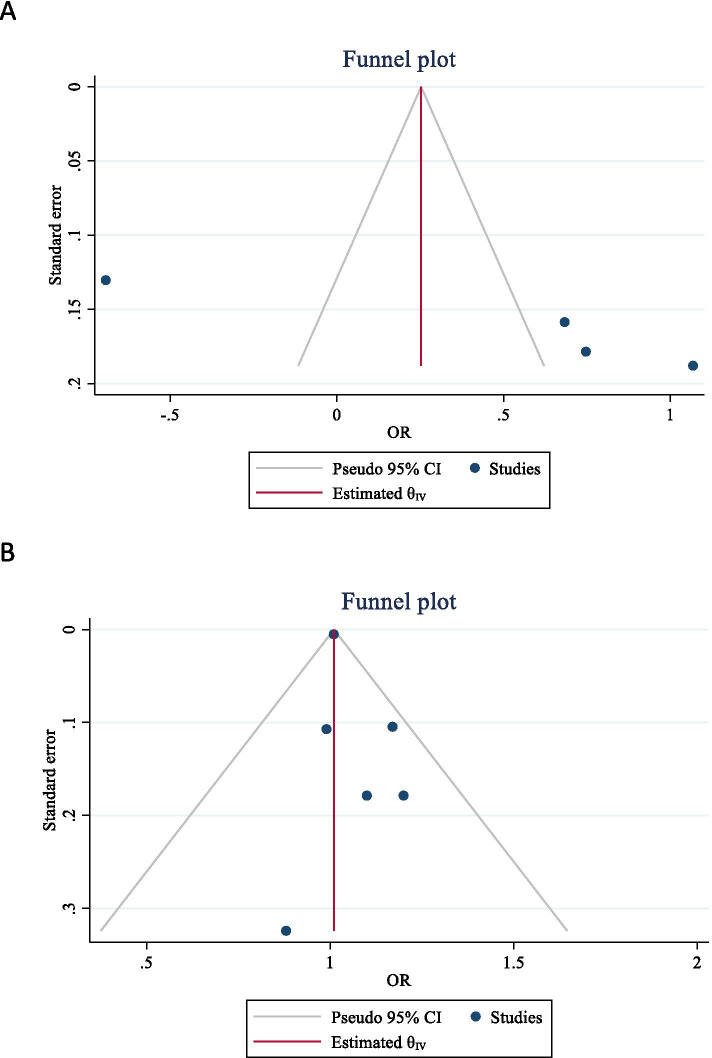
The funnel plot of the obesity effect on the risk of Helicobacter pylori infection (**A**) and the effect of Helicobacter pylori on obesity (**B**)

Because zero was not in the 95% confidence interval of the Egger’s test, significant bias occurred in the publication of the results related to the effect of obesity on the risk of H. pylori infection (Egger’s test = 4.10, *P* = 0.003, 95% CI: 2.68 to 5.52). The funnel plot of the pooled effect of obesity on the risk of H. pylori infection was shown in Fig. 4(A). Also no significant bias occurred in the publication of the results of the H. pylori effect on the development of obesity (Egger’s test = 0.437, *P* = 0.265, 95% CI: -0.50 to 1.37). The funnel plot of the pooled effect of H. pylori on the development of obesity was shown in Fig. 4(B).

## Discussion

This study was the first meta-analysis investigating the correlation between H. pylori infection and obesity worldwide. The pooled analysis of 8 case–control studies demonstrated that the H. pylori prevalence in the obese population was higher than that in the normal population and also, H. pylori positive individuals were more likely to be obese than H. pylori negative individuals. Therefore, there was a positive correlation between the prevalence of obesity and the risk of H. pylori infection around the world.

The underlying mechanisms of these results are not well-recognized; however, some potential pathways explain these findings. First, regulation of the gastric hormones (e.g. ghrelin and leptin) deals with the energy hemostasis [32]. The ghrelin secretion by P/D1 cells of stomach in hunger status increases appetite and induces food intake while the leptin secretion by P cells of stomach and adipocytes following food intake, decreases appetite and induces satiety [33, 34]. H. pylori positive individuals have lower plasma levels of ghrelin and leptin than non-infected individuals. Therefore, lower serum leptin delays the feeling of satiety during eating and leads to more energy intake and develops obesity. Meanwhile, the lower serum ghrelin in H. pylori-infected patients indicates lower production of this hormone due to atrophic gastritis as well as the physiological response of the body to positive energy balance in the obese population [35–37]. In addition, it is revealed that the cure of H. pylori infection increases the serum ghrelin level [38]. The second potential factor that explains the positive relationship between the risk of obesity and H. pylori infection is the higher insulin resistance in H. pylori positive patients, that leads to obesity development [39]. The third factor that potentially interferes with our findings, is the impairment of the immune function of the gastrointestinal tract among the obese population. Accordingly, the monocytes of the immune system have a low ability of maturation into macrophages [40–42] and also, the natural killer cells of obese individuals have less cytotoxic activity than those of healthy-BMI individuals, that both result in less bactericidal activity, higher susceptibility of H. pylori survival and the higher prevalence of H. pylori infection in obese subjects [43].

The meta-analysis by Xu et al. [44] in 2019 revealed that H. pylori infection increased the risk of obesity development among the Chinese population with an odds ratio of 1.20 (95% CI: 1.13 to 1.28). This relationship was congruent with our findings. It has been also reported that H. pylori positive patients had higher total cholesterol, triglyceride, and LDL levels and a lower HDL level [44].

In this meta-analysis, the results showed that the effect of Helicobacter pylori infection on the occurrence of obesity after a combination of studies controlling confounding variables was 1.01 with a confidence interval of 1.01 to 1.03. It could be argued that there was almost no association between the presence of infection and the occurrence of obesity because the size of the association was not very significant. Based on the diagnosis method of Helicobacter pylori infection, the results showed that, after combining the results of studies which had used the ELIZA method to detect bacteria, the effect size was 1.11 with a confidence interval of 0.99 to 1.25, indicating that people with infection were 11% more obese than people who did not have an infection. This relationship was close to a significant level. In this subgroup analysis, the heterogeneity rate in all was close to 0%, which indicated the absence of heterogeneity in these analyzes and studies. In determining the relationship between obesity and the incidence of Helicobacter pylori infection, after combining the results of case studies that diagnosed the infection using the histology and biopsy, the odds ratio was 1.14, but was not statistically significant and the amount of heterogeneity decreased relative to the overall findings. This indicated that different diagnostic methods to determine the association between obesity and infection were a major source of heterogeneity and its creation in the overall combination of studies.

## Limitations

One of the limitations of this study was the limited number of articles that did not allow the investigation of the relationship based on important variables. In addition, the most important limitation of this study was the lack of isolation of Helicobacter pylori genotypes in meta-analysis studies. Also, the type of Helicobacter pylori strains was not specified in the studies. For example, Helicobacter pylori type I is more involved in the occurrence of metabolic disorders than the type II. If these cases were identified, the authors could perform subgroup analysis in this study based on these variables and could compare the effects. Obesity is also a disorder that is strongly influenced by the lifestyle and genetic or hereditary factors. It is suitable to conduct a more detailed study in the future in the form of cohort studies with an appropriate sample size in order to determine this effect by precisely controlling the confounding variables.

## Conclusion

The prevalence of H. pylori infection in obese individuals was higher than that in the healthy-BMI population and also the risk of obesity development in H. pylori-positive patients was higher than that in non-infected participants.

## Data Availability

All relevant data are within the manuscript and its supporting information files.
